# Supplementary material for: Phenotypic characterization and analysis of complete genomes of two distinct strains of the proposed species “L. swaminathanii”
Source: Sci Rep. 2022 Jun 1;12:9137. doi: 10.1038/s41598-022-13119-y (PMC9159981; doi:10.1038/s41598-022-13119-y)
Supplement: Supplementary file 1 — Supplementary Figure S1. [file 41598_2022_13119_MOESM1_ESM.pdf]

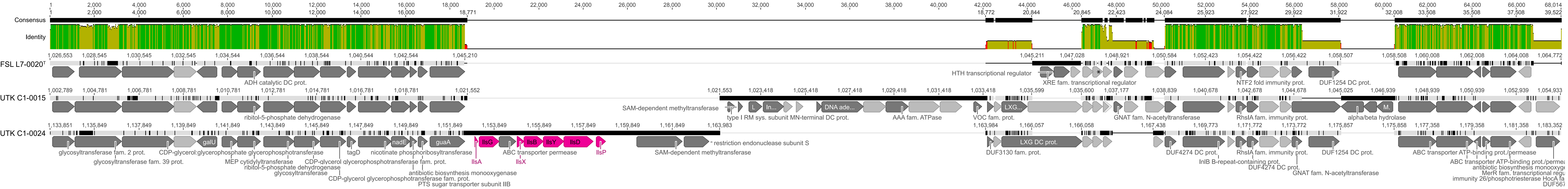

Supplementary Figure S1. Listeria pathogenicity island 3 (LIPI-3) in UTK C1-0024

Region of the whole-genome alignment (Figure 1) that contains LIPI-3 (CDS in pink). CDS annotated as hypothetical proteins are light gray and all others are dark gray.
